# Hyoid Bone Position in Relation to Vertical Skeletal Pattern: A Systematic Review and Meta-Analysis

**DOI:** 10.3390/diagnostics16172826

**Published:** 2026-09-02

**Authors:** Davinia Pérez-Sánchez, Marta Ibor-Miguel, Juan Ignacio Aura-Tormos, Laura Marqués-Martínez, Clara Guinot-Barona, Esther García-Miralles

**Affiliations:** 1 Dentistry Department, Faculty of Medicine and Health Sciences, Catholic University of Valencia San Vicente Mártir, 46001 Valencia, Spain; davinia.perez@ucv.es (D.P.-S.); marta.ibor@ucv.es (M.I.-M.); laura.marques@ucv.es (L.M.-M.); 2 Doctoral School, Faculty of Medicine and Health Sciences, Catholic University of Valencia, 46001 Valencia, Spain; 3 Faculty of Medicine and Dentistry, University of Valencia, 46010 Valencia, Spain; juan.aura@uv.es (J.I.A.-T.); m.esther.garcia@uv.es (E.G.-M.)

**Keywords:** hyoid bone, vertical skeletal pattern, hyperdivergent, cephalometrics, systematic review, meta-analysis, airway

## Abstract

**Objectives**: This study aimed to determine whether hyoid bone position differs across vertical skeletal growth patterns through a systematic review and meta-analysis using cephalometric and CBCT measurements. **Methods**: Four electronic databases were searched up to June 4, 2026. Twenty-three reports were included in the qualitative synthesis (22 comparative studies and one normative reference report; 3404 participants), and 22 comparative studies underwent NIH quality assessment. Six studies contributed to the H-MP meta-analysis. For H-C3, four studies reporting vertical-pattern contrasts formed the primary pool, although residual sagittal confounding remained; two sagittal-proxy studies were added only in the sensitivity analysis. **Results**: For H-MP, pooled SMD was 0.185 (95% CI: −0.231 to 0.601; *p* = 0.383; I^2^ = 87.2%; k = 6). For H-C3, the primary pooled MD was −1.30 mm (95% CI: −2.32 to −0.28; *p* = 0.013; I^2^ = 48.6%; k = 4); the sensitivity pool yielded −1.45 mm (95% CI: −2.51 to −0.38; *p* = 0.008; k = 6). **Conclusions**: H-MP did not differ significantly between patterns. The pooled H-C3 estimate indicated a more posterior hyoid position in groups classified as hyperdivergent; however, residual sagittal confounding prevents its attribution to vertical divergence alone. Cranial-base or coordinate-based measurements independent of mandibular-plane inclination should be prioritised.

## 1. Introduction

The hyoid bone occupies a unique anatomical position in the craniofacial complex: it is the only bone in the head and neck region that lacks bony articulations with adjacent structures. Suspended by a muscular–ligamentous system connecting it to the mandible, skull base, pharynx, larynx, and cervical spine, the hyoid bone plays a central role in deglutition, phonation, and airway patency. Because its position is dynamically determined by the relative tension of the supra- and infrahyoid muscle groups, it has long been considered an anatomical indicator of the functional and morphological equilibrium of the oropharyngeal complex.

The relationship between hyoid bone position and craniofacial morphology has attracted substantial attention in orthodontic research [1,2]. From a clinical standpoint, understanding how vertical skeletal growth patterns—hyperdivergent, normodivergent, and hypodivergent—relate to hyoid positioning has implications for orthodontic treatment planning, airway assessment, and prediction of post-treatment stability. Hyperdivergent patients, characterised by a steep mandibular plane angle, clockwise-rotated mandible, and long anterior facial height, are known to exhibit narrower pharyngeal airway dimensions and altered tongue posture. Whether these morphological characteristics are consistently associated with changes in hyoid bone position, however, remains unresolved.

Early cephalometric studies established normative values for the hyoid triangle [1]—a geometric construct relating the hyoid to the third cervical vertebra (C3) and the mandibular symphysis (retrognathion)—and proposed that the hyoid position is maintained in close conjunction with adjacent anatomical structures regardless of skeletal pattern [2]. Subsequent investigations, however, reported conflicting findings: some observed a more inferior and posterior hyoid position in hyperdivergent patients, while others found no significant differences across groups. These discrepancies may reflect methodological heterogeneity, including differences in reference planes, imaging modalities (two-dimensional cephalometry versus cone-beam computed tomography), patient age groups, and classification criteria for vertical skeletal pattern.

To date, no systematic review with meta-analysis has synthesised the available evidence on hyoid bone position across vertical skeletal growth patterns. Given the increasing availability of three-dimensional imaging and the growing number of published studies, a quantitative synthesis is both timely and necessary. The present systematic review and meta-analysis aimed to determine whether hyoid bone position—assessed by the distance from the hyoid to the mandibular plane (H-MP) and the distance from the hyoid to the third cervical vertebra (H-C3)—differs significantly between hyperdivergent, normodivergent, and hypodivergent individuals.

## 2. Materials and Methods

### 2.1. Protocol and Registration

This systematic review was conducted in accordance with the Preferred Reporting Items for Systematic Reviews and Meta-Analyses (PRISMA 2020) guidelines [3]. The review protocol was registered in the PROSPERO international prospective register (CRD420261416928; registered on 7 June 2026).

### 2.2. Eligibility Criteria

Studies were eligible if they: (1) included human subjects with complete permanent or near-complete dentition; (2) compared hyoid bone position between at least two vertical skeletal growth patterns or provided a normative reference dataset used to interpret a directly comparable hyoid measurement; (3) reported cephalometric or CBCT measurements of hyoid bone position; and (4) were published as original research articles in peer-reviewed journals or academic theses. No restrictions were applied regarding age, sex, ethnicity, language, or publication year. Comparative studies were excluded if the comparator was the breathing pattern, malocclusion class exclusively, or treatment type rather than vertical skeletal pattern, or if relevant numerical data were unavailable.

### 2.3. Information Sources and Search Strategy

Four electronic databases were searched on 4 June 2026: MEDLINE via PubMed (*n* = 74), Web of Science (*n* = 112), Scopus (*n* = 110), and Embase (*n* = 40). The search strategy combined terms related to the hyoid bone (“hyoid bone”, “hyoidale”, “os hyoideum”) and vertical skeletal pattern (“vertical pattern”, “hyperdivergent”, “hypodivergent”, “facial type”, “growth pattern”, “mandibular plane”, “open bite”, “deep bite”, “long face syndrome”). No date or language restrictions were applied. Reference lists of included studies and relevant reviews were screened manually.

### 2.4. Study Selection

Records were deduplicated and managed using Rayyan (Rayyan Systems Inc., Cambridge, MA, USA). Two reviewers (D.P.S. and M.I.M.) independently screened titles and abstracts, followed by full-text evaluation of potentially eligible studies. Disagreements were resolved by consensus with a third reviewer (E.G.M.). Studies not retrievable after a comprehensive search (*n* = 3) were recorded as unavailable.

### 2.5. Data Extraction

Data were extracted independently by two reviewers (D.P.S. and E.G.M.) using a pre-piloted standardised form. Extracted variables included: author and year, country, study design, sample size and demographic characteristics, imaging modality, cephalometric software, method of vertical pattern classification, hyoid bone measurements (type, landmark definitions, values), statistical methods, and key findings.

### 2.6. Risk of Bias Assessment

Risk of bias was evaluated using the NIH Quality Assessment Tool for Observational Cohort and Cross-Sectional Studies [4], comprising 14 items. Each item was rated Yes, No, Partial, Not applicable, or Cannot be determined. To ensure reproducibility, an operational scoring rule was applied: Yes = 1 point; Partial = 0.5 points; and No/Cannot be determined = 0 points. Not applicable items were excluded from the denominator. Overall quality was rated Good at ≥75% of the maximum applicable score, Fair at 50–74%, and Poor at <50%. These thresholds were adopted as an explicit operational rule because NIH guidance does not prescribe numerical cut-offs. Q7 and Q13 were treated as not applicable in cross-sectional designs. Pae 2008 [5] was assessed as a longitudinal cohort; Kumar 2017 [6] was classified as a pre/post-treatment longitudinal observational study, for which temporal and follow-up items remained applicable. Bibby and Preston 1981 [1] provided normative reference values rather than an exposure-group comparison and was therefore not included in the comparative-study risk-of-bias summary.

### 2.7. Statistical Analysis

The primary outcomes were: (1) H-MP, the perpendicular distance from the hyoidale to the mandibular plane, and (2) H-C3, the linear distance from the hyoidale to the most inferior-anterior point of the third cervical vertebra. For H-MP, the standardised mean difference (Hedges g) was used because of variations in measurement technique. For H-C3, the mean difference in millimetres was used given greater methodological consistency. Positive SMD for H-MP indicates a more inferior hyoid position in hyperdivergent subjects; negative MD for H-C3 indicates a more posterior position.

Random-effects meta-analyses were performed using the DerSimonian–Laird method [7]. Heterogeneity was assessed by the Q statistic (Cochrane), the I^2^ statistic, and the between-study standard deviation τ. Sensitivity analyses included: (a) exclusion of Erdinc 2003 [8], which presented an unusually high standard deviation in the normodivergent group, and (b) an H-C3 analysis extending the primary pool of Kocakara 2022 [9], Tarkar 2016 [10], Sivakumar 2017 [11], and Karandish 2026 [12] with Mortazavi 2018 [13] and Özen 2026 [14]. The four primary studies reported vertical-pattern contrasts but retained residual sagittal confounding; the two additional studies principally used sagittal grouping. All calculations were performed in Python 3.11. A *p*-value < 0.05 was considered statistically significant. Studies were eligible for quantitative synthesis if they reported means and standard deviations for at least two comparable groups and provided extractable data for H-MP or H-C3; studies rated Poor for risk of bias and those reporting only medians were retained in the narrative synthesis only. Formal subgroup analysis and meta-regression were not performed because the number of studies per outcome (k = 4–6) was below the commonly recommended minimum of k ≥ 10 [15]. Formal assessment of publication bias (funnel plot and Egger’s test) was not performed for the same reason; this limitation is acknowledged in the Discussion.

The primary H-C3 analysis comprised four studies reporting a vertical-pattern contrast (Kocakara 2022 [9], Tarkar 2016 [10], Sivakumar 2017 [11], and Karandish 2026 [12]). This restriction reduced but did not eliminate sagittal confounding because Sivakumar 2017 [11] and Karandish 2026 [12] also incorporated Class II versus Class I contrasts. Mortazavi 2018 [13] and Özen 2026 [14], whose principal grouping was sagittal, were added only in a sensitivity analysis, producing an extended pool of six studies.

### 2.8. Use of Generative AI

During the preparation of this manuscript, the authors used Claude (Anthropic, San Francisco, CA, USA) to assist with language editing and statistical code debugging. All data extraction and statistical analyses were performed and independently verified by the authors. The authors reviewed and edited all AI-assisted output and take full responsibility for the content of the manuscript.

## 3. Results

### 3.1. Study Selection

**Figure 1:**
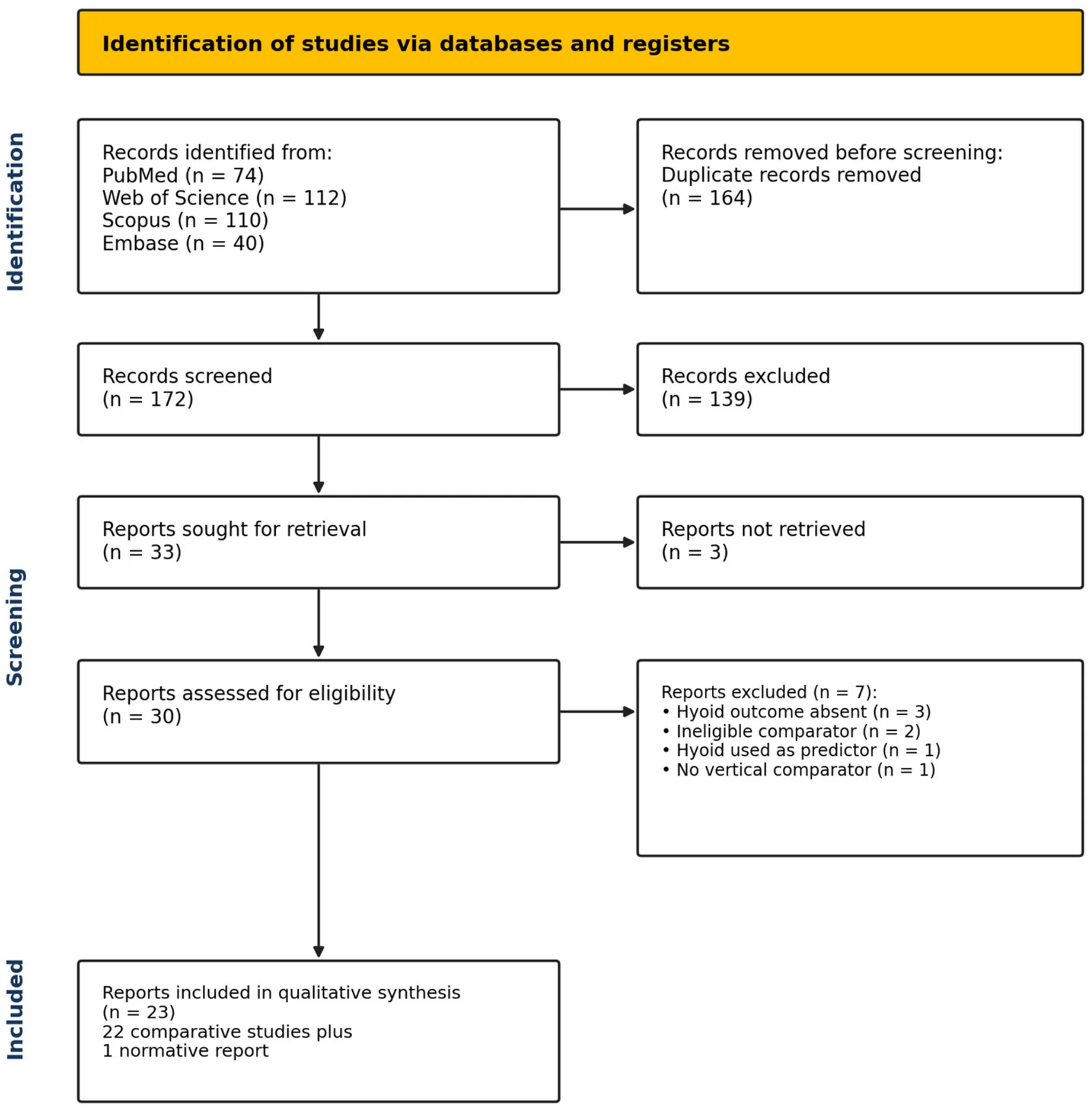
PRISMA 2020 flow diagram illustrating the study selection process.

The electronic search retrieved 336 records (PubMed: 74; Web of Science: 112; Scopus: 110; Embase: 40) (Figure 1). After removal of 164 duplicates, 172 unique records were screened and 139 were excluded. Thirty-three reports were sought for retrieval; three could not be retrieved, leaving 30 reports assessed for eligibility. Seven were excluded after full-text review: three did not measure hyoid position as an outcome (Zhong 2010 [16]; Ucar 2011 [17]; Sana 2025 [18]), two used an ineligible comparator (Ucar 2012 [19]; Sayınsu 2006 [20]), one used hyoid position as a predictor rather than an outcome (Haskell 2014 [21]), and one lacked a vertical comparator group (Li 2017 [22]). Twenty-three reports were included in the qualitative synthesis: 22 comparative studies and one normative reference report. Six studies contributed to the H-MP meta-analysis. Four studies reporting a vertical-pattern contrast formed the primary H-C3 pool, although residual sagittal confounding remained; two additional predominantly sagittal-proxy studies were included only in sensitivity analysis.

### 3.2. Study Characteristics

The 23 included reports were published between 1981 and 2026 and encompassed 3404 participants. Twenty comparative studies were cross-sectional; Pae 2008 [5] was a prospective longitudinal cohort with 15-year follow-up; and Kumar 2017 [6] used a longitudinal pre/post-treatment observational design. Bibby and Preston 1981 [1] supplied normative C3-H values. Seventeen reports used conventional two-dimensional lateral cephalometry, five used CBCT, and one combined both modalities. Vertical classification most commonly used FMA, SN-GoGn, or the Jarabak ratio. Detailed characteristics are presented in Table 1.

### 3.3. Risk of Bias

**Figure 2:**
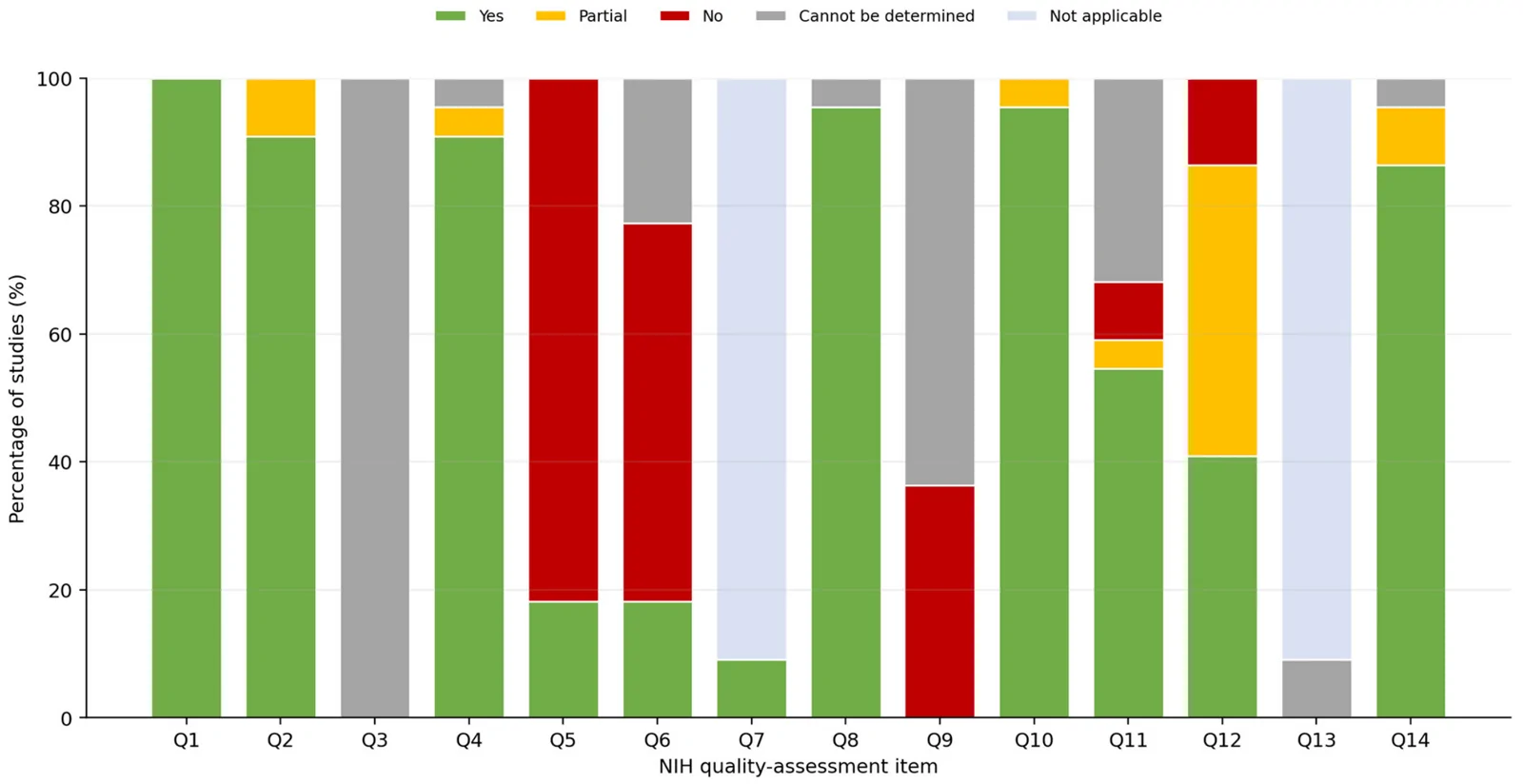
Distribution of NIH item ratings across the 22 comparative studies. S, Yes; P, Partial; N, No; ND, Cannot be determined; NA, Not applicable.

Among the 22 comparative studies assessed, the recalculated overall ratings are reported in Table S1. Eighteen studies lacked an a priori sample-size calculation; assessor blinding was absent or only partially documented in 13. Participation rate (Q3) was rated Cannot be determined when reports omitted the number of eligible or invited individuals. Exposure assessment (Q9) was frequently rated No or Cannot be determined because validation, reproducibility, or consistent implementation of skeletal-pattern classification was insufficiently reported. These recurring weaknesses reduce confidence that the pooled estimates are free from selection, measurement, and small-study bias. Domain-level results and the full-item key are provided in Table S1 and summarised in Figure 2.

### 3.4. Narrative Synthesis

#### 3.4.1. Vertical Position of the Hyoid Bone (H-MP)

Of the 23 included reports, 14 reported H-MP or an equivalent vertical measurement. Findings were heterogeneous. Large or methodologically stronger studies, including Kocakara 2022 [9], Pae 2008 [5], and Sivakumar 2017 [11], generally reported no significant between-pattern difference. Kumar 2017 [6] likewise found no significant difference in hyoid position between normodivergent and hyperdivergent groups following treatment. By contrast, Erdinc 2003 [8], Jung 2015 [25], and Ghazala 2023 [31] reported significant differences, although direction and measurement definitions varied.

#### 3.4.2. Anteroposterior Position of the Hyoid Bone (H-C3)

H-C3 was reported in 12 studies. Smaller H-C3 values were commonly observed in skeletal Class II or hyperdivergent groups, but sagittal and vertical classifications were frequently intertwined. Mortazavi 2018 [13] and Özen 2026 [14] were therefore treated as sagittal-proxy studies and excluded from the primary pool of studies reporting a vertical-pattern contrast. Kocakara 2022 [9] found no significant vertical-pattern difference. Bibby and Preston [1] provided the normative C3-H reference value of 31.76 ± 2.9 mm.

#### 3.4.3. Additional Measurements

Several studies used alternative measurements. Al-Somairi 2024 [33] (CBCT, *n* = 368) reported a significantly greater H-VP (hyoid to vertical plane) in hypodivergent subjects, suggesting a more anterior position. Pae 2008 [5], the prospective longitudinal cohort study, found that HYRGN (hyoid to retrognathion) was significantly larger in brachyfacial (hypodivergent) subjects at the baseline, and that changes in hyoid position over 15 years were significantly greater in dolichofacial subjects. Kang 2025 [35], using multivariate regression in 185 CBCT scans, found that horizontal hyoid position was associated with sagittal—but not vertical—skeletal pattern after adjustment for both dimensions simultaneously.

### 3.5. Meta-Analysis

#### 3.5.1. H-MP—Hyperdivergent vs. Normodivergent

Six studies contributed data to the H-MP meta-analysis. The pooled SMD was 0.185 (95% CI: −0.231 to 0.601; *p* = 0.383), indicating no statistically significant difference between hyperdivergent and normodivergent subjects. Heterogeneity was very high (I^2^ = 87.2%; Q = 39.10, df = 5, *p* < 0.001; τ = 0.475). In the sensitivity analysis excluding Erdinc 2003 [8] (unusual SD in the normodivergent group), the pooled SMD was 0.301 (95% CI: −0.123 to 0.725; *p* = 0.165), remaining non-significant with similar heterogeneity (I^2^ = 87.0%).

#### 3.5.2. H-C3—Hyperdivergent vs. Normodivergent

**Figure 3:**
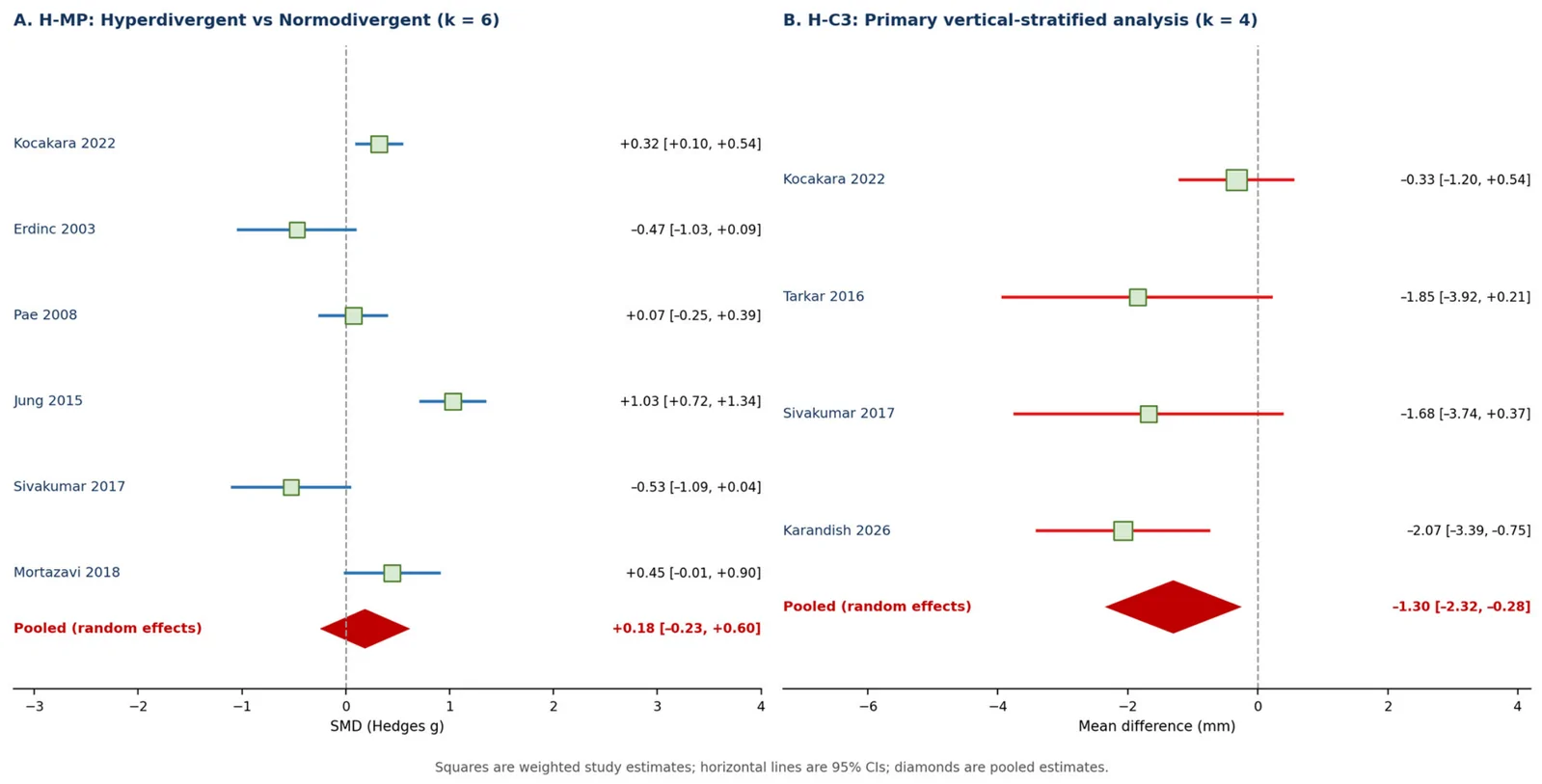
Forest plots of hyoid bone position using DerSimonian–Laird random-effects models: (**A**) H-MP standardised mean difference; (**B**) H-C3 mean difference for the primary four-study analysis of studies reporting a vertical-pattern contrast. Residual sagittal confounding remained. The extended six-study H-C3 sensitivity estimate is reported in Table 2. Study labels correspond to the following references, using the same numerical citation format as in the text: (**A**) Kocakara 2022 [9]; Erdinc 2003 [8]; Pae 2008 [5]; Jung 2015 [25]; Sivakumar 2017 [11]; Mortazavi 2018 [13]. (**B**) Kocakara 2022 [9]; Tarkar 2016 [10]; Sivakumar 2017 [11]; Karandish 2026 [12].

Four studies reporting H-C3 contrasts involving vertical divergence contributed to the primary H-C3 meta-analysis (Kocakara 2022 [9], Tarkar 2016 [10], Sivakumar 2017 [11], Karandish 2026 [12]). The pooled MD was −1.30 mm (95% CI: −2.32 to −0.28; *p* = 0.013; I^2^ = 48.6%; Q = 5.84, df = 3; τ = 0.717), indicating a more posterior hyoid position relative to C3 in groups classified as hyperdivergent. This estimate cannot be attributed to vertical divergence alone because residual sagittal confounding remained in the primary pool. A sensitivity analysis extending the pool to include two studies using sagittal classification as a proxy (Mortazavi 2018 [13], Özen 2026 [14]; k = 6) yielded a pooled MD of −1.45 mm (95% CI: −2.51 to −0.38; *p* = 0.008; I^2^ = 60.8%; τ = 1.002). The direction and magnitude were similar, but the extended analysis did not resolve sagittal confounding. Meta-analysis results are presented in Table 2 and Figure 3.

## 4. Discussion

This systematic review and meta-analysis are, to our knowledge, the first to quantitatively synthesise evidence on hyoid bone position across vertical skeletal growth patterns. The main findings can be summarised as follows: (1) the vertical position of the hyoid bone (H-MP) did not differ significantly between hyperdivergent and normodivergent individuals in meta-analysis, with substantial heterogeneity among studies, and (2) the pooled H-C3 estimate indicated a more posterior position in groups classified as hyperdivergent. The H-C3 association remained similar in sensitivity analysis, but residual sagittal confounding prevents its attribution to vertical divergence alone.

The absence of a significant pooled effect for H-MP, despite reports in the literature of a lower hyoid position in hyperdivergent patients, deserves careful interpretation [5,8,9,25,31]. The very high heterogeneity (I^2^ = 87.2%) indicates that the between-study variance exceeds what would be expected from sampling error alone, suggesting important methodological sources of variability. Critically, H-MP is measured as the perpendicular distance from the hyoidale to the mandibular plane—a plane that is, by definition, steeper in hyperdivergent subjects [33]. This creates a geometric confound: even if the hyoid bone occupies a similar absolute position in space, a steeper mandibular plane will yield a smaller H-MP in hyperdivergent patients, potentially masking a true difference. This observation, consistent with the findings of Kocakara et al. [9] in the largest study (*n* = 611), raises fundamental concerns about the validity of H-MP as a comparative measure across groups with different mandibular inclinations.

Future comparative studies should avoid H-MP as the principal vertical outcome because the mandibular plane is part of the phenotype used to define divergence. Cranial-base references or true Cartesian/three-dimensional coordinate systems independent of mandibular-plane inclination are methodologically preferable and would reduce geometric coupling between group definition and outcome measurement.

In contrast, H-C3—which relates the hyoid to the cervical spine rather than to the mandible—is not directly determined by mandibular-plane inclination and may therefore be less susceptible to this specific geometric confound. The primary pooled MD was −1.30 mm (k = 4) [9,10,11,12], and all four-point estimates were negative; however, three individual confidence intervals crossed the null result, and two studies incorporated sagittal Class II versus Class I contrasts. The pooled association should therefore not be interpreted as proof of a robust or independent vertical-pattern effect. One possible explanation is that clockwise mandibular rotation and accompanying changes in tongue and suprahyoid muscle position could contribute to posterior hyoid displacement, but this mechanism remains hypothetical in the context of the included evidence. Opdebeeck et al. [2] described relevant craniofacial rotational patterns, although the present findings do not establish a causal pathway.

The discordant result from Kocakara 2022 [9] (which found no significant difference in H-C3 in a paediatric sample) warrants specific comment. With the largest sample (*n* = 611) and a factorial design controlling simultaneously for sagittal and vertical pattern, this study had adequate statistical power to detect even small differences. Its null result in a paediatric population may reflect genuine age-dependent differences: the relationship between skeletal pattern and hyoid position may consolidate during growth completion, consistent with the longitudinal data of Pae 2008 [5], who showed that hyoid descent over 15 years was significantly greater in dolichofacial adults.

An important finding from the narrative synthesis is that the anteroposterior dimension of hyoid position appears more sensitive to vertical pattern than the vertical dimension. Multiple studies using different reference systems converge on this conclusion: Sivakumar 2017 [11] reported significant C3-H differences between Class II hyperdivergent and Class I normodivergent subjects, attributing the effect to mandibular retrognathism rather than facial divergence per se; Mortazavi 2018 [13] reported smaller H-C3 in Class II than Class I; and Karandish 2026 [12], with a 3 × 3 factorial design (*n* = 296), demonstrated consistently smaller C3-H in Class II across all vertical subgroups. These converging findings suggest that sagittal and vertical components jointly determine hyoid anteroposterior position, with the Class II pattern—particularly when combined with hyperdivergent morphology—producing the most posterior hyoid positions.

Kang 2025 [35], employing multivariate linear regression in CBCT images, provided the most methodologically sophisticated analysis: controlling simultaneously for sagittal, vertical, and transverse dimensions, only the sagittal pattern (ANB and APDI) was independently associated with horizontal hyoid position. The vertical pattern (ODI and SN-GoGn) was not. This finding challenges the interpretation of studies that report vertical-pattern effects without controlling for the sagittal pattern and highlights the need for factorial designs or regression-based approaches in future research.

Several limitations must be acknowledged. First, substantial heterogeneity in reference planes, classification criteria, imaging modality, and age-restricted quantitative synthesis. Second, most studies used two-dimensional cephalometry. Third, predominantly cross-sectional designs preclude causal inference. Fourth, sagittal discrepancies were often uncontrolled and may substantially distort anteroposterior hyoid measurements; Class II retrognathism can alter mandibular and cervical relationships independently of vertical morphology. Kang et al. [35] showed in a simultaneous multivariable model that the horizontal hyoid position was associated with a sagittal, but not vertical, skeletal pattern, directly cautioning against attributing the pooled H-C3 association solely to vertical divergence. Fifth, 18 of the 22 assessed studies lacked an a priori sample-size calculation and assessor blinding was absent or only partially documented in 13, increasing imprecision and measurement-bias concerns. Sixth, Q3 and Q9 were poorly reported across the evidence base, limiting assessment of selection and exposure-classification validity. Finally, publication bias could not be assessed reliably because each meta-analysis included fewer than 10 studies; the pooled estimates remain vulnerable to small-study effects.

The clinical relevance of the observed anteroposterior difference warrants explicit consideration. A pooled MD of −1.3 mm in H-C3 is modest in absolute terms. In the context of the normative values reported by Bibby and Preston [1], whose standard deviation for C3-H was 2.9 mm, this displacement corresponds to approximately 0.45 standard deviations. Nevertheless, a clinically meaningful threshold has not been established, and the pooled estimate should not be interpreted as evidence of airway compromise or functional impairment. Future studies should correlate hyoid position with outcomes such as airway resistance and swallowing efficiency.

From a clinical perspective, orthodontists should not rely on H-MP alone to compare hyoid position across growth patterns because it depends geometrically on mandibular-plane inclination. H-C3 may provide a less mandibular-plane-dependent anteroposterior measure, but its validity as an indicator of vertical skeletal pattern remains uncertain because of sagittal confounding. Future studies should use standardised measurements and reference systems independent of mandibular inclination. Three-dimensional imaging should be reserved for situations in which it is clinically justified and should not be recommended solely for research measurement when an adequate lower-dose method is available.

## 5. Conclusions

Based on the available evidence from 23 reports (22 comparative studies and one normative reference report; *n* = 3404 participants), the following conclusions can be drawn:

1. The vertical position of the hyoid bone, measured as H-MP, did not differ significantly between hyperdivergent and normodivergent subjects in meta-analysis (SMD = 0.185; 95% CI: −0.231 to 0.601; *p* = 0.383), with very high heterogeneity (I^2^ = 87.2%). This may be partly attributable to the geometric dependence of H-MP on mandibular-plane inclination.
2. The pooled H-C3 estimate indicated a more posterior hyoid position in groups classified as hyperdivergent (MD = −1.30 mm; 95% CI: −2.32 to −0.28; *p* = 0.013), with a similar estimate in the sensitivity analysis. Residual sagittal confounding prevents attribution of this association to vertical divergence alone, and its clinical significance remains uncertain.
3. The effects of vertical and sagittal skeletal patterns on hyoid position are not independent; factorial and multivariate designs are needed to disentangle their contributions.
4. Future studies should standardise hyoid measurement methods and use reference systems independent of mandibular inclination. Three-dimensional imaging should be used when clinically justified.

## Figures and Tables

**Table 1:** Characteristics of included studies.

First Author (Year)	Country	*n*	Age (y)	Design	Image	Classification	Key Outcomes
Haralabakis (1993) [23]	Greece	82	15–27	CS	Ceph	Open bite vs. CL I	H-PP ↑; H-Po-FH ↓ in OB
Erdinc (2003) [8]	Turkey	75	~12	CS	Ceph	SN-GoGn + Jarabak	H-SN, H-FH, H-PP ↓ in hyper ***
Pae (2008) [5]	USA	163	46–61	Cohort	Ceph	FMA	HYRGN hypo > normo > doli ***
Jena (2011) [24]	India	71	15–25	CS	Ceph	FMA + SN-MP	H-PTRper: hypo > normo ***
Jung (2015) [25]	Korea	182	~25	CS	Ceph	Björk sum	H-MP: skeletal > dental pattern ***
Tarkar (2016) [10]	India	90	18–32	CS	Ceph	Jarabak + FMA	H-RGn hypo > normo * (Bibby)
Kumar (2017) [6]	India	40	20–27	Pre/post observational	Ceph	FMA	No between-pattern difference; hyoid moved posteriorly/inferiorly after treatment
Sivakumar (2017) [11]	India	100	18–25	CS	Ceph	ANB + FMA	C3H: CII hyper < CI normo **
Mortazavi (2018) [13]	Iran	110	≥18	CS	Ceph	ANB	H-C3: CI > CII *; H-PP: CI > CIII *
Ribeiro (2018) [26]	Portugal	31	NR	CS	Ceph	Overbite	H-MP vs. Ricketts norms *
Nejaim (2018) [27]	Brazil	161	21–58	CS	CBCT	VERT index + ANB	LL hyoid: doli > meso (morphology)
Chen (2021) [28]	China	90	~16	CS	CBCT	ANB + MP-FH	H-Me: hypo CL I > normo/hyper ***
Soyoye (2021) [29]	Nigeria	174	Adults	CS	Ceph	Overbite	C3-Hy: reduced > normal > deep *
Kocakara (2022) [9]	Turkey	611	≤17	CS	Ceph	ANB + SN-GoGn	All hyoid measures NS
Guo (2022) [30]	China	160	20–50	CS	CBCT	ANB + SN-MP	H-X: CII hyper < all others **
Ghazala (2023) [31]	Pakistan	75	Adults	CS	Ceph	SN-MP	H-MP, H-axis-MP: hyper > normo *
Mackevičiūtė (2025) [32]	Lithuania	60	Adults	CS	Ceph	Overbite in CII	C3-RGn: open > deep bite *
Al-Somairi (2024) [33]	China	368	Adults	CS	CBCT	FMA	H-VP: hypo > normo > hyper ***
Karandish (2026) [12]	Iran	296	Adults	CS	Ceph	ANB + FMA	C3-H, H-RGn: CII < CI ***
Özen (2026) [14]	Turkey	120	18–30	CS	Ceph	ANB	H-RGn: CIII > CI, CII *
Domenyuk (2025) [34]	Russia	106	Adults	CS	Ceph + CBCT	Vertical/neutral/horizontal	H-Me: horizontal > others * (median)
Kang (2025) [35]	Korea	185	~29	CS	CBCT	Continuous (ANB, ODI, SN-GoGn)	Hyoid x: class III (sagittal) only
Bibby & Preston (1981) [1] †	S. Africa	54	~13	CS	Ceph	CL I (normative)	Normative values: C3-H = 31.8 ± 2.9 mm

CS, cross-sectional; Ceph, lateral cephalometry; CBCT, cone-beam computed tomography; NS, not significant; ↑, increased; ↓, decreased. * *p* < 0.05; ** *p* ≤ 0.01; *** *p* ≤ 0.001. † Bibby and Preston [1] provided normative values and were not included in the comparative-study NIH assessment.

**Table 2:** Meta-analysis results.

Study	k	Effect (95% CI)	CI low	CI High	*p*	I^2^ (%)	τ	RoB	Sig.
ANALYSIS 1: H-MP—Hyperdivergent vs. Normodivergent (SMD, Hedges g; positive = hyoid more inferior in hyperdivergent)									
Kocakara 2022 [9]		0.322 (0.105, 0.538)	0.105	0.538				GOOD	
Erdinc 2003 [8] †		−0.472 (−1.034, 0.090)	−1.034	0.090				FAIR	
Pae 2008 [5]		0.073 (−0.248, 0.393)	−0.248	0.393				FAIR	
Jung 2015 [25]		1.031 (0.721, 1.341)	0.721	1.341				FAIR	
Sivakumar 2017 [11]		−0.527 (−1.091, 0.036)	−1.091	0.036				FAIR	
Mortazavi 2018 [13]		0.448 (−0.005, 0.901)	−0.005	0.901				FAIR	
POOLED (RE)	6	0.185 (−0.231, 0.601)	−0.231	0.601	0.383	87.2	0.475	—	NS
PRIMARY ANALYSIS: H-C3—Studies reporting a vertical-pattern contrast (residual sagittal confounding remains)									
Kocakara 2022 [9]		−0.33 (−1.20, 0.54)	−1.20	0.54				GOOD	
Tarkar 2016 [10]		−1.85 (−3.92, 0.21)	−3.92	0.21				FAIR	
Sivakumar 2017 [11]		−1.68 (−3.74, 0.37)	−3.74	0.37				FAIR	
Karandish 2026 [12]		−2.07 (−3.39, −0.75)	−3.39	−0.75				FAIR	
POOLED (RE)	4	−1.30 (−2.32, −0.28)	−2.32	−0.28	0.013	48.6	0.717	—	*
SENSITIVITY ANALYSIS: H-C3—Extended pool including two predominantly sagittal-proxy studies									
Kocakara 2022 [9]		−0.33 (−1.20, 0.54)	−1.20	0.54				GOOD	
Tarkar 2016 [10]		−1.85 (−3.92, 0.21)	−3.92	0.21				FAIR	
Sivakumar 2017 [11]		−1.68 (−3.74, 0.37)	−3.74	0.37				FAIR	
Mortazavi 2018 [13]		−3.54 (−5.61, −1.47)	−5.61	−1.47				FAIR	
Karandish 2026 [12]		−2.07 (−3.39, −0.75)	−3.39	−0.75				FAIR	
Özen 2026 [14]		0.25 (−1.86, 2.36)	−1.86	2.36				GOOD	
POOLED (RE)	6	−1.45 (−2.51, −0.38)	−2.51	−0.38	0.008	60.8	1.002	—	**

RE, random-effects model (DerSimonian–Laird). SMD, standardised mean difference (Hedges g). MD, mean difference (mm). Positive SMD = hyoid more inferior in hyperdivergent. Negative MD = hyoid more posterior in hyperdivergent. RoB: GOOD/FAIR/POOR (NIH Tool). CI, confidence interval; † High SD in normodivergent group; * *p* < 0.05; ** *p* < 0.01; NS, not significant.

## Data Availability

The data supporting the conclusions of this article are derived from publicly available published studies. The meta-analysis dataset and extraction forms are available from the corresponding author upon reasonable request.

## Supplementary Materials

The following supporting information can be downloaded at https://www.mdpi.com/article/10.3390/diagnostics16172826/s1, Table S1: Risk of bias assessment (NIH Quality Assessment Tool). Table S2: PRISMA 2020 Checklist.
