# Supplementary material for: Hyoid Bone Position in Relation to Vertical Skeletal Pattern: A Systematic Review and Meta-Analysis
Source: Diagnostics (Basel). 2026 Sep 2;16(17):2826. doi: 10.3390/diagnostics16172826 (PMC13565066; doi:10.3390/diagnostics16172826)
Supplement: Supplementary file 1 [file diagnostics-16-02826-s001.zip › diagnostics-4483171_Supplementary_Table_S1_FINAL (2).pdf]

Table S1. Risk-of-bias assessment using the NIH Quality Assessment Tool

Q1–Q14 are items of the NIH Quality Assessment Tool for Observational Cohort and Cross-Sectional Studies. S = Yes; N = No; P = Partial; NA = Not applicable; ND = Cannot be determined. Overall ratings were calculated using an explicit operational rule: S = 1 point, P = 0.5 points, and N/ND = 0 points; NA items were excluded from the denominator. Good corresponded to ≥75% of the maximum applicable score, Fair to 50–74%, and Poor to <50%. These thresholds are an operational rule adopted for transparency; the NIH guidance does not prescribe numerical cut-offs.

| Study | Q1 | Q2 | Q3 | Q4 | Q5 | Q6 | Q7 | Q8 | Q9 | Q10 | Q11 | Q12 | Q13 | Q14 | Overall |
|---|---|---|---|---|---|---|---|---|---|---|---|---|---|---|---|
| Haralabakis 1993 [23] | S | S | ND | S | N | N | NA | S | N | S | S | N | NA | S | FAIR |
| Erdinc 2003 [8] | S | S | ND | S | N | N | NA | S | N | S | S | P | NA | S | FAIR |
| Pae 2008 [5] | S | S | ND | S | N | N | S | S | N | S | P | S | ND | S | FAIR |
| Jena 2011 [24] | S | S | ND | S | N | N | NA | S | N | P | ND | P | NA | S | FAIR |
| Jung 2015 [25] | S | S | ND | S | N | S | NA | S | N | S | S | P | NA | S | FAIR |
| Tarkar 2016 [10] | S | S | ND | S | N | N | NA | S | N | S | N | P | NA | S | FAIR |
| Sivakumar 2017 [11] | S | S | ND | S | N | N | NA | S | ND | S | S | S | NA | S | FAIR |
| Mortazavi 2018 [13] | S | S | ND | S | N | N | NA | S | N | S | S | S | NA | S | FAIR |
| Nejaim 2018 [27] | S | S | ND | S | N | N | NA | S | N | S | S | P | NA | S | FAIR |
| Ribeiro 2018 [26] | S | P | ND | ND | N | N | NA | S | ND | S | ND | N | NA | P | POOR |
| Soyoye 2021 [29] | S | S | ND | S | N | ND | NA | S | ND | S | ND | P | NA | S | FAIR |
| Chen 2021 [28] | S | S | ND | S | N | N | NA | S | ND | S | S | S | NA | S | FAIR |
| Kocakara 2022 [9] | S | S | ND | S | S | ND | NA | S | ND | S | S | S | NA | S | GOOD |
| Ghazala 2023 [31] | S | S | ND | S | S | N | NA | S | ND | S | N | N | NA | S | FAIR |
| Al-Somairi 2024 [33] | S | S | ND | S | N | S | NA | S | ND | S | S | S | NA | S | GOOD |
| Mackevičiūtė 2025 [32] | S | S | ND | S | N | N | NA | S | ND | S | ND | P | NA | S | FAIR |
| Domenyuk 2025 [34] | S | S | ND | S | N | ND | NA | S | ND | S | ND | P | NA | P | FAIR |
| Kang 2025 [35] | S | S | ND | P | N | N | NA | S | ND | S | S | S | NA | S | FAIR |
| Kumar 2017 [6] | S | P | ND | S | N | S | S | ND | ND | S | ND | P | ND | ND | POOR |

| Study | Q1 | Q2 | Q3 | Q4 | Q5 | Q6 | Q7 | Q8 | Q9 | Q10 | Q11 | Q12 | Q13 | Q14 | Overall |
|---|---|---|---|---|---|---|---|---|---|---|---|---|---|---|---|
| Guo 2022 [30] | S | S | ND | S | N | ND | NA | S | ND | S | S | S | NA | S | FAIR |
| Karandish 2026 [12] | S | S | ND | S | S | ND | NA | S | ND | S | ND | S | NA | S | FAIR |
| Özen 2026 [14] | S | S | ND | S | S | S | NA | S | ND | S | S | P | NA | S | GOOD |

**NIH item key**

Q1. Was the research question or objective clearly stated?
Q2. Was the study population clearly specified and defined?
Q3. Was the participation rate of eligible persons at least 50%?
Q4. Were all subjects selected from the same or similar populations and were inclusion/exclusion criteria prespecified and applied uniformly?
Q5. Was a sample-size justification, power description, or variance/effect estimate provided?
Q6. For analyses, were the exposure(s) of interest measured before the outcome(s)?
Q7. Was the timeframe sufficient to reasonably expect an association between exposure and outcome?
Q8. For exposures varying in amount or level, did the study examine different exposure levels in relation to the outcome?
Q9. Were exposure measures clearly defined, valid, reliable, and implemented consistently?
Q10. Was exposure assessed more than once over time?
Q11. Were outcome measures clearly defined, valid, reliable, and implemented consistently?
Q12. Were outcome assessors blinded to participants' exposure status?
Q13. Was loss to follow-up after baseline 20% or less?
Q14. Were key potential confounding variables measured and adjusted statistically?

**Interpretive notes**

Q3 was rated ND when the original report did not provide both the number eligible or invited and the number enrolled, preventing calculation of a participation proportion.

Q7 and Q13 were rated NA for cross-sectional studies because temporal follow-up and attrition were not part of those designs. They were retained as applicable items for the longitudinal studies.

Q9 concerns the validity and reliability of the exposure classification (vertical or sagittal skeletal pattern). N or ND indicates that validation, reproducibility, or consistent implementation of the classification could not be confirmed from the report; it does not indicate that hyoid outcome measurement was absent.
