# Supplementary material for: Hyoid Bone Position in Relation to Vertical Skeletal Pattern: A Systematic Review and Meta-Analysis
Source: Diagnostics (Basel). 2026 Sep 2;16(17):2826. doi: 10.3390/diagnostics16172826 (PMC13565066; doi:10.3390/diagnostics16172826)
Supplement: Supplementary file 1 [file diagnostics-16-02826-s001.zip › diagnostics-4483171_Supplementary_Table_S2_PRISMA_and_Methods_FINAL.pdf]

TABLE S2. PRISMA 2020 CHECKLIST

Hyoid Bone Position in Relation to Vertical Skeletal Pattern: A Systematic Review and Meta-Analysis

Pérez-Sánchez D, Ibor-Miguel M, Aura-Tormos JI, Marqués-Martínez L, Guinot-Barona C, García-Miralles E

Updated against the final 13-page manuscript and the corrected supplementary search/methods file.

#	PRISMA 2020 item	Location	Status	Remarks
**TITLE**				
1	Title: identify the report as a systematic review.	Title (p. 1)	Complete	The title identifies the report as a systematic review and meta-analysis.
**ABSTRACT**				
2	Structured abstract (see PRISMA 2020 for Abstracts checklist).	Abstract (p. 1)	Complete	The abstract reports the objectives, methods, principal results and conclusions.
**INTRODUCTION**				
3	Rationale for the review in the context of existing knowledge.	Introduction (pp. 1-2)	Complete	Rationale and conflicting evidence are described.
4	Explicit statement of objectives or questions.	Introduction, final paragraph (p. 2)	Complete	The objective is explicit.
**METHODS**				
5	Eligibility criteria and grouping for the syntheses.	Section 2.2 (p. 2)	Complete	Eligibility and exclusion criteria are reported.
6	Information sources and date last searched.	Section 2.3 (p. 3)	Complete	Four databases searched on 4 June 2026; reference lists also screened.
7	Full search strategies for all databases, including filters and limits.	Suppl. Methods A	Complete	Complete strategies are provided for PubMed, Embase, Web of Science and Scopus.
8	Study selection process, including reviewers and independence.	Section 2.4 (p. 3)	Complete	Two independent reviewers; disagreements resolved with a third reviewer.
9	Data collection process, including reviewers and independence.	Section 2.5 (p. 3)	Complete	Independent extraction by two reviewers using a pre-piloted form.
10a	Outcomes for which data were sought.	Sections 2.5 and 2.7 (p. 3)	Complete	H-MP and H-C3 are defined.
10b	Other variables for which data were sought.	Section 2.5 (p. 3)	Complete	Study and measurement variables are listed.

11	Methods used to assess risk of bias, including reviewers and independence.	Section 2.6 (p. 3); Suppl. Methods B.1	Complete	NIH tool, scoring, thresholds and independent assessment process are reported.
12	Effect measures used for each outcome.	Section 2.7 (pp. 3-4)	Complete	Hedges g for H-MP; mean difference in millimetres for H-C3.
13a	Processes used to decide which studies were eligible for each synthesis.	Section 2.7 (p. 4); Suppl. Methods B.2	Complete	Eligibility for meta-analysis is specified.
13b	Methods required to prepare data for presentation or synthesis.	Section 2.7 (p. 4); Suppl. Methods B.3	Complete	Handling of medians and unavailable data is described.
13c	Methods used to tabulate or visually display results.	Tables 1-2; Figures 1-3 (pp. 5-9)	Complete	Study characteristics, risk of bias and meta-analysis results are displayed.
13d	Methods used to synthesise results.	Section 2.7 (pp. 3-4)	Complete	DerSimonian-Laird random-effects models in Python 3.11.
13e	Methods used to explore possible causes of heterogeneity.	Section 2.7 (p. 4); Suppl. Methods B.4	Complete	No subgroup/meta-regression because k < 10; sensitivity and narrative exploration described.
13f	Sensitivity analyses conducted to assess robustness.	Sections 2.7 and 3.5 (pp. 4, 8-9)	Complete	Both sensitivity analyses are reported.
14	Methods used to assess risk of bias due to missing results.	Section 2.7 (p. 4); Suppl. Methods B.5	Complete	Formal tests were not performed because k < 10, and the rationale is explicitly reported.
15	Methods used to assess certainty in the body of evidence.	Suppl. Methods B.7; Sections 2.6-2.7 and Discussion	Complete	A formal GRADE framework was not considered necessary for the review objective and scope. Confidence was assessed narratively using risk of bias, heterogeneity, imprecision, residual confounding, sensitivity analyses, and potential small-study effects.
**RESULTS**				
16a	Results of the search and selection process, ideally with a flow diagram.	Section 3.1 (p. 4); Figure 1 (p. 5)	Complete	Selection counts and flow diagram are reported.
16b	Studies that appeared eligible but were excluded, with reasons.	Section 3.1 (p. 4)	Complete	Three unavailable and seven excluded reports are described.
17	Characteristics of each included study.	Section 3.2 (p. 5); Table 1 (p. 6)	Complete	All 23 included reports are tabulated.
18	Risk-of-bias assessments for each included study.	Section 3.3 (p. 7); Supplementary Table S1	Complete	Item-level NIH ratings are provided for 22 comparative studies.
19	Results for all outcomes for each study.	Section 3.4 (pp. 7-8); Table 2 and Figure 3 (pp. 8-9)	Complete	Narrative and study-level quantitative results are reported.
20a	Characteristics and risk of bias among studies contributing to each synthesis.	Sections 3.3-3.5 (pp. 7-9); Tables 1-2	Complete	Characteristics and NIH ratings are reported.

20b	Results of all statistical syntheses.	Section 3.5; Table 2; Figure 3 (pp. 8-9)	Complete	Effects, confidence intervals and heterogeneity are reported.
20c	Results of investigations of possible causes of heterogeneity.	Sections 3.5.1-3.5.2 (pp. 8-9)	Complete	Heterogeneity statistics and the limits on subgroup analysis are reported.
20d	Results of all sensitivity analyses.	Sections 3.5.1-3.5.2; Table 2 (pp. 8-9)	Complete	Both sensitivity analyses are reported.
21	Assessments of risk of bias due to missing results for each synthesis.	Sections 2.7 and 4 (pp. 4, 11); Suppl. Methods B.5	Complete	Formal assessment was not performed because k < 10; this is explicitly reported and acknowledged as a limitation.
22	Assessments of certainty in the body of evidence for each outcome.	Suppl. Methods B.7; Discussion and Conclusions	Complete	Confidence in both primary outcomes was assessed narratively through risk of bias, heterogeneity, imprecision, residual confounding, sensitivity analyses, and potential small-study effects; no formal GRADE category was assigned.
**DISCUSSION**				
23a	General interpretation of results in the context of other evidence.	Section 4 (pp. 9-11)	Complete	Findings are interpreted in context.
23b	Limitations of the evidence included in the review.	Section 4 (p. 11)	Complete	Heterogeneity, design, imaging, confounding and bias concerns are discussed.
23c	Limitations of the review processes used.	Section 4 (p. 11)	Partial	Small-study and publication-bias limitations are discussed; review-process limitations are not a distinct comprehensive subsection.
23d	Implications for practice, policy and future research.	Section 4 (p. 11); Section 5 (pp. 11-12)	Complete	Clinical and research implications are provided.
**OTHER INFORMATION**				
24a	Registration information, including register name and number.	Section 2.1 (p. 2)	Complete	PROSPERO CRD420261416928; registration date reported.
24b	Where the review protocol can be accessed.	Section 2.1 (p. 2); Suppl. Methods B.6	Complete	The PROSPERO record is identified; data and forms are available on request.
24c	Amendments to registration or protocol.	Not reported	Not reported	No amendments are reported.
25	Sources of support and role of funders or sponsors.	Funding (p. 12)	Complete	No external funding.
26	Competing interests of review authors.	Conflicts of Interest (p. 12)	Complete	No conflicts declared.
27	Availability of data, forms, code and other materials.	Data Availability Statement (p. 12)	Complete	The meta-analysis dataset and extraction forms are available from the corresponding author upon reasonable request.

SUPPLEMENTARY INFORMATION

Hyoid Bone Position in Relation to Vertical Skeletal Pattern: A Systematic Review and Meta-Analysis

Pérez-Sánchez D, Ibor-Miguel M, Aura-Tormos JI, Marqués-Martínez L, Guinot-Barona C, García-Miralles E

This file contains the complete search strategies and additional methodological clarifications consistent with the final manuscript.

Part A. Complete Search Strategies by Database

All searches were conducted on 4 June 2026 with no date or language restrictions. The record counts below match the four primary databases reported in the final manuscript (total records retrieved: 336).

A.1 PubMed / MEDLINE

Interface: PubMed Advanced Search Builder. Retrieved records: n = 74.

```
("hyoid bone"[MeSH Terms] OR "hyoid bone"[tiab] OR "hyoidale"[tiab] OR "os hyoideum"[tiab])
AND
("vertical pattern"[tiab] OR "hyperdivergent"[tiab] OR "hypodivergent"[tiab] OR "facial type"[tiab] OR "growth pattern"[tiab] OR "mandibular
plane"[tiab] OR "open bite"[tiab] OR "deep bite"[tiab] OR "long face syndrome"[tiab])
```

A.2 Embase

Interface: Embase.com Advanced Search. Retrieved records: n = 40.

```
('hyoid bone'/exp OR 'hyoid bone':ti,ab OR 'hyoidale':ti,ab OR 'os hyoideum':ti,ab)
AND
('vertical pattern':ti,ab OR 'hyperdivergent':ti,ab OR 'hypodivergent':ti,ab OR 'facial type':ti,ab OR 'growth pattern':ti,ab OR 'mandibular
plane':ti,ab OR 'open bite':ti,ab OR 'deep bite':ti,ab OR 'long face syndrome':ti,ab)
```

A.3 Web of Science Core Collection

Interface: Web of Science Advanced Search. TS = Topic. Retrieved records: n = 112.

```
TS=("hyoid bone" OR "hyoidale" OR "os hyoideum")
AND
TS=("vertical pattern" OR "hyperdivergent" OR "hypodivergent" OR "facial type" OR "growth pattern" OR "mandibular plane" OR "open bite" OR "deep bite"
OR "long face syndrome")
```

A.4 Scopus

Interface: Scopus Advanced Search. TITLE-ABS-KEY searches title, abstract and keywords. Retrieved records: n = 110.

```
TITLE-ABS-KEY("hyoid bone" OR "hyoidale" OR "os hyoideum")
AND
TITLE-ABS-KEY("vertical pattern" OR "hyperdivergent" OR "hypodivergent" OR "facial type" OR "growth pattern" OR "mandibular plane" OR "open bite" OR
"deep bite" OR "long face syndrome")
```

Part B. Additional Methods Clarifications

B.1 Risk-of-bias assessment independence (PRISMA Item 11)

Risk of bias was assessed independently by two reviewers (D.P.S. and E.G.M.) using the NIH Quality Assessment Tool for Observational Cohort and Cross-Sectional Studies. Disagreements were resolved by consensus with a third reviewer (C.G.-B.). The item ratings and overall classifications follow the explicit operational scoring rule reported in Section 2.6 of the final manuscript and in Supplementary Table S1: Yes = 1 point; Partial = 0.5 points; No/Cannot be determined = 0 points; Not applicable items were excluded from the denominator. Overall quality was rated Good at >=75%, Fair at 50-74%, and Poor at <50% of the maximum applicable score.

B.2 Eligibility for quantitative synthesis (PRISMA Item 13a)

Studies were eligible for meta-analysis if they reported means and standard deviations for at least two comparable groups, provided extractable data for H-MP or H-C3, and were not rated Poor in the risk-of-bias assessment. Studies reporting only medians or insufficient group-level data were retained in the narrative synthesis only.

B.3 Handling of missing or non-standard data (PRISMA Item 13b)

Domenyuk 2025 reported results as medians and was retained in the narrative synthesis but excluded from meta-analysis. No missing standard deviations were imputed. Three reports could not be retrieved in full text and were recorded as unavailable; their eligibility for quantitative synthesis could not be assessed.

B.4 Exploration of heterogeneity (PRISMA Item 13e)

Formal subgroup analysis and meta-regression were not performed because the number of studies per outcome (k = 4-6) was below the commonly recommended minimum of k >= 10. Heterogeneity was examined through the prespecified sensitivity analyses and narrative comparison of study characteristics, including age, imaging modality and skeletal-pattern classification criteria.

B.5 Assessment of publication bias (PRISMA Items 14 and 21)

Funnel-plot asymmetry and Egger's test were not performed because each meta-analysis included fewer than 10 studies. The possibility of publication bias could not therefore be formally excluded and is acknowledged as a limitation in the final manuscript.

B.6 Protocol registration and access (PRISMA Item 24b)

The review protocol is registered in PROSPERO (CRD420261416928; registered on 7 June 2026). The registration number identifies the protocol record. The review data and extraction forms are available from the corresponding author upon reasonable request, as stated in the final manuscript.

B.7 Assessment of confidence in the findings (PRISMA Items 15 and 22)

A formal GRADE certainty-of-evidence framework was not considered necessary for the objective and scope of this review. Confidence in the findings was assessed narratively by considering study-level risk of bias, inconsistency and heterogeneity, imprecision of the pooled estimates, residual sagittal confounding, the results of sensitivity analyses, and the potential for small-study and publication-bias effects. These considerations are reported in the Results, Discussion, and Conclusions; no formal GRADE category was assigned.
